# Association Between Upper Limb Impairment and Function Within One Month Post Stroke and Self-care at Six Months

**Published:** 2025-09-30

**Authors:** Jigna Patel, Qinyin Qiu, Gerard G Fluet, Holly Gorin, Jennifer Gutterman, Kiran Karunakaran, Karen J Nolan, Emma Kaplan, Alma S Merians, Sergei V Adamovich

**Affiliations:** 1 Department of Rehabilitation and Movement Sciences, School of Health Professions Rutgers, The State University of New Jersey, Newark, New Jersey, USA; 2 Department of Biomedical Engineering, New Jersey Institute of Technology, University Heights, Newark New Jersey, USA; 3 Kessler Foundation, West Orange, New Jersey, USA

**Keywords:** Stroke, CVA, Upper limb, Quality of life, Biomarkers, Subacute, Virtual reality

## Abstract

**Introduction:**

Stroke leaves many people with upper limb impairments which significantly affect function and Quality Of Life (QOL). Most studies report outcomes at the impairment and activity level. Less attention has been paid to real-life use of the affected upper limb in daily activities. Self-reported measures of performance of activity in daily life using the affected upper limb is important to obtain as people may have minimal deficits on impairment and activity level outcomes yet still have significant difficulties performing self-care and other Activities of Daily Living (ADLs). These difficulties have an important impact on QOL. Discovering easy clinical measures that are associated with the ability to perform ADLs in the long-term is vital as this can lead to more effective care planning by providers, more realistic expectations for survivors, and more efficient allocation of time and resources.

**Objective:**

This exploratory study investigated the association between affected upper limb movements from the Upper Extremity Fugl-Meyer Assessment (UEFMA) as well as grip and grasp items from the Action Research Arm Test (ARAT) measured within thirty days post stroke and the self-care domain of the EuroQol-5D-5L measured at six months post stroke. These higher-level affected arm movements require some degree of corticospinal tract function which is a known predictor of functional outcome.

**Methods:**

Baseline clinical and six-month EuroQol-5D-5L data were obtained from sixty participants who completed a randomized clinical trial that evaluated robotically facilitated virtual reality for the upper limb in the subacute period post stroke. Cross tabs were used to quantify the association between the baseline ability to perform higher level affected arm movements and the ability to wash and dress oneself at six months post stroke using the self-rated EuroQol-5D-5L health related QOL measure.

**Results:**

The ability to perform higher level affected arm movements that require some degree of corticospinal tract function within thirty days post stroke was significantly associated with self-care at six months post stroke in a group of moderate to severely impaired individuals with stroke.

**Discussion:**

We propose easy to assess hand, wrist, and arm movements that may be effective clinical tools to project independence with self-care in the long term in similar populations.

## INTRODUCTION

Stroke is a leading cause of disability in adults in the United States (US) [1]. Upper limb impairments are common post stroke, and more than two thirds of those initially affected continue to live with long-term functional limitations which impact their independence and quality of life [2]. Numerous studies have evaluated a variety of biomarkers to predict motor outcomes [3]. The ability to accurately predict long term functional outcomes and real life use of the affected arm from easy to measure variables obtained early after stroke can lead to more effective care planning by rehabilitation providers, more realistic expectations for stroke survivors, and more efficient allocation of time and resources [4].

Upper extremity function plays an important role in the ability to perform activities of daily living thus affecting health related Quality Of Life (QOL) [5]. Most studies report outcomes at the impairment and activity level [6]. Less attention has been paid to real-life use of the affected upper limb in daily activities [6]. Self-reported measures of performance of activity in daily life using the affected upper limb is important to obtain as people may have minimal deficits on impairment and activity level outcomes yet still have significant difficulties performing self-care and other Activities of Daily Living (ADLs) [7]. A commonly used tool to evaluate QOL in the stroke population is the EuroQol-5D-5L [8]. This is a self-rated QOL measure where individuals rate themselves on the ability to complete five different domains of QOL, for example, mobility and self-care. The difficulty performing each measure is rated as no problem (score of 1) to extreme problem/unable to perform (score of 5).

The purpose of this study was to explore the quantitative association between affected active hand, wrist and non-synergistic arm movements from the Upper Extremity Fugl-Meyer (UEFMA) as well as grip and grasp items from the Action Research Arm Test (ARAT) measured within 30 days after stroke onset and the self-care domain of the EuroQol-5D-5L measured at six months post stroke. Both the UEFMA and the ARAT are widely used, reliable and valid outcome measures used in the stroke population [9,10]. We surmised that the ability to actively perform isolated wrist and hand movements on the affected side, as well as non-synergistic and functional arm movements with the affected upper extremity (movements that require some degree of Corticospinal Tract function (CST)) less than one month post stroke would be associated with higher levels of activity as measured by the EuroQol-5D-5L self-care domain at six months post lesion. CST integrity is an established predictor of upper limb motor recovery after stroke [11].

The population was a subgroup with severe to moderate impairment post stroke, who participated in a randomized clinical trial that evaluated robotically facilitated Virtual Reality (VR) rehabilitation of the upper limb in the subacute period post stroke [12].

## METHODS

### Study design

This was an exploratory sub analysis from a Randomized Controlled trial (RCT) with four parallel arms that was completed in 2024 [12]. The RCT was approved by the Internal Review Boards (IRBs) of the Kessler Foundation, Rutgers University, and the New Jersey Institute of Technology (NJIT). All research was performed in accordance with relevant guidelines and regulations set by the IRBs. The trial was posted on 6/26/2018 (registration number: NCT03569059) at ClinicalTrials.gov prior to participant recruitment. The clinical results and details about the randomization process from the RCT [12].

### Participants

This exploratory study included sixty participants who were part of the three treatment groups in the RCT. The three groups included an Early VR group (EVR), a Delayed VR group (DVR), and a Dose Matched Usual Care group (DMUC). The methods section includes details about these three groups. We chose to include only participants who had received research training to standardize the dose of training received by all people used for this sub analysis. The RCT showed no difference in any clinical outcome between any of the training groups across all five time points [12]. Refer to the supplementary table for ANOVAs showing no difference between the three treatment groups at baseline and at six months post stroke on the UEFMA and the ARAT. Participants for the RCT were recruited from a large inpatient rehabilitation center. The RCT included people who: 1) had a diagnosed stroke (ischemic or hemorrhagic) less than 30 days prior to study initiation, 2) were between 30–95 years of age, 3) were able to follow instructions, 4) had severe to moderate arm weakness (≥ 10/66 and ≤ 49/66 on the UEFMA 19), and 5) had intact cutaneous sensation. Individuals with UEFMA scores <10 were not included as they would not have had the motor ability to utilize the VR and robotic systems. Potential participants were excluded from the RCT if they: 1) were not independent prior to the stroke, 2) were too ill to tolerate training, 3) had persistent motor impairment from a prior stroke, 4) had aphasia or spatial neglect precluding their performing the tasks or following task instructions-assessed by their inpatient therapists during their initial evaluations, 5) had ≥ 1 on the NIHSS limb ataxia item, 6) had severe proprioceptive loss, 7) scored ≥ 3 on the Modified Ashworth Scale (for the elbow, wrist, or finger flexors), or 8) had a previous medical history of neurological deficits or orthopedic conditions that limited their affected arm and hand movement.

### Interventions

The sixty participants received either VR/robotic training (Early Training Group (EVR), Delayed Training Group (DVR) or Dose Matched Usual Care (DMUC) initiated at various times post stroke.

**EVR group:** received usual care therapy plus an extra 10 1-hour sessions of intensive upper limb therapy focusing on the hand using robotically facilitated rehabilitation interventions presented in non-immersive virtual environments and initiated 5–30 days post-stroke. The systems utilized included the NJIT RAVR, NJIT Track Glove and the NJIT HoVRS to train the affected upper extremity individually (or bilaterally with the unaffected upper extremity) and included activities such as using individual fingers to play a virtual piano, extending fingers to hit a virtual ball, transporting the arm to eliminate virtual spaceships, using a pinch grasp to move a virtual object onto higher and higher levels, reaching to lift virtual cups onto a haptic shelf placed at a variety of heights, integrating reach and hand movements to manipulate fruit from virtual trees, integrating reach and forearm pronation and supination to hammer a virtual nail into a piece of wood, and using both arms to play virtual ping pong. Details about the NJIT RAVR and NJIT Track Glove systems can be found in [13]. This training was provided initially while the participants were inpatients at Kessler Institute for Rehabilitation (5–30 days post stroke). Subjects completed training on an outpatient basis after discharge from inpatient rehabilitation [12].

**DVR group:** received usual care therapy plus an extra 10 1-hour sessions of the same training as the EVR group, initiated 31–60 days post-stroke. Some DVR group subjects started training as inpatients, but most of the subjects in this group performed all training on an outpatient basis [12].

**DMUC group:** received usual care plus 10 1-hour sessions of adaptive and progressive task and impairment based physical and occupational therapy, including strengthening and ROM for the affected upper limb, provided by trained study physical or occupational therapists. This was provided initially to participants while they were inpatients (5–30 days post stroke). Subjects completed training on an outpatient basis after discharge from inpatient rehabilitation [12].

### Outcome measures

**Baseline:** Baseline clinical outcomes utilized in this sub analysis included the UEFMA (impairment level outcome measure) and the activity level outcome measure. They were obtained prior to thirty days post stroke.

Upper Extremity Fugl-Meyer Assessment (UEFMA) [14]. This is an impairment based upper extremity measure consisting of 33 movements that test single and multi-joint movement in and out of synergy, digit individuation, speed, dysmetria, ataxia, and reflexes. Each item is rated on a three-point scale: 0=cannot perform, 1=performs partially, 2=performs fully, for a total score of 66. Higher scores indicate less impairment and more isolated active movement ability. For this sub analysis, all seven hand tasks were utilized. Although in humans the Reticulospinal Tract (RST) assists with gross hand movements such as mass grasp, all active hand movements require some degree of Corticospinal Tract Integrity (CST) and thus should be associated with greater overall recovery [11,15,16]. Additionally, the task of active wrist flexion extension with the elbow straight and forearm pronation/supination with the elbow straight were used in this analysis as they require higher level non-synergistic active movement that combines motion at the shoulder, elbow, forearm and wrist. In humans, the RST supplies large muscle groups in a synergistic manner and thus would not contribute greatly to such tasks [17]. We hypothesized that higher-level tasks that require some degree of CST function would be better able to categorize motor function between the participants at baseline.

Action Research Arm Test (ARAT) [18]. This is a 19-item test with four subscales that measure the following upper extremity functional tasks at the ICF activity level: (grasp (6 items), grip (4 items), pinch (6 items) and movement (3 items). Each item is rated on a four-point scale with the following values, 0=no movement, 1=the movement task is partially performed, 2=the movement task is completed but takes abnormally long, and 3=normal movement. Scores range from 0 to 57 with higher scores indicating better motor function. For this sub analysis the task requiring grasp of a 2.5 cm cube and placing it on top of a thirty-four cm shelf and the task requiring lifting a large tube off a peg and placing it on another peg thirty cm from the first peg were included. They were chosen as they are the easiest tasks included in the ARAT that require a combination of shoulder, elbow, wrist, and hand movement. These tasks also require some degree of CST function [17].

**Self-care outcome:** EuroQol-5D-5L [8]. This is a self-rated QOL outcome measure. Participants rated themselves on five measures: 1) mobility (ambulation), 2) self-care (bathing and dressing), 3) usual activities (their current function in “work, study, housework, family or leisure activities”), 4) pain/discomfort, and 5) anxiety/depression. We utilized the self-care domain rated at six months post stroke to assess how the ability to move the affected hand and arm within thirty days post stroke is associated with the long-term ability to perform ADLs. See (Table 1) for the values and associated descriptions of the self-care domain scores.

**Statistical methods:** Cross tabs with Fisher’s Exact Test were used to determine the association between the preselected initial hand and arm movements measured within thirty days post stroke (baseline UEFMA and ARAT) and the self-care domain on the EuroQol-5D-5L measured at six months post stroke [19]. As this was an exploratory study, a cross tab was performed for each hand or arm movement. A standardized residual greater than 1.96 or less than −1.96 was used to determine a significant association between the two variables for each query. Cramer’s V was used to represent the strength of each association. Statistics were conducted using SPSS version 31.

Scores of three, four, or five on the self-care domain were collapsed into one score of three as we did not have enough participants with scores of four or five at six months for an adequate distribution [20]. Thus, a score of three means the person required moderate or greater assistance for self-care.

## RESULTS

Fifty nine of the sixty participants completed both the baseline and six-month testing sessions. One participant missed the six-month session so their four-month data which was part of the RCT was used for their six-month values. The average age of participants was 63.12 (SD=10.99) and the mean days post stroke at baseline was 16.03 (SD=6.37). Based on their impairment level at baseline using the UEFMA, the majority of participants were moderately impaired [21]. Please refer to (Table 2) for the group’s baseline data as well as the itemized scores and percentages at six months for the self-care domain of the EuroQol-5D-5L.

### Associations

A score of 2 on the UEFMA meaning the ability to actively and fully: 1) extend or flex the affected fingers, 2) touch the affected index finger with the thumb, and 3) flex or extend the affected wrist with the elbow straight within the first thirty days past post stroke demonstrated a statistically significant, moderate association with independence with washing and dressing oneself at six months post stroke as measured by the self-care domain on the EuroQol-5D-5L. Conversely, a score of 0 on the UEFMA or ARAT meaning no ability at all to: 1) actively extend the affected fingers, 2) actively grip and move a large tube, 3) actively grasp and move a 2.5 cm cube, or 4) actively pronate/supinate the forearm with the elbow straight within thirty days post stroke demonstrated a statistically significant, moderate association with requiring moderate or more assistance at six months post stroke with washing and dressing oneself as tested by the self-care domain on the EuroQol-5D-5L. Please refer to (Table 3) for the proportion of individuals who scored the listed score per outcome item with their score on the self-care domain of the EuroQol-5D-5L at six months. The corresponding p value for the Fisher’s Exact test, as well as Cramer’s V and p values for each query are also provided. Unless reported in (Table 3), all other scores on the UEFMA and ARAT showed a non-significant relationship with the EuroQol-5D-5L self-care score.

## DISCUSSION

The study is unique because it quantitatively assessed the association between specific items from the UEFMA and ARAT that require some CST function with the domain of self-care on the EuroQol-5L-5D [15–17]. Self-reported measures of self-care provide a more realistic view of real world use of the affected arm. This exploratory sub analysis included people with moderate to severe stroke who completed ten extra hours of either VR/robotic training or dose matched usual care in the early subacute period. This was in addition to the usual care they received which started at a state-of-the-art inpatient rehabilitation facility followed by either homecare or outpatient therapy. The ability to fully actively extend or flex the affected fingers, touch the affected index finger actively and fully with the thumb and actively flex or extend the affected wrist with the elbow straight within the first thirty days past stroke demonstrated a statistically significant, moderate association with independence with washing and dressing oneself at six months post stroke as measured by the self-care domain on the EuroQol-5D-5L. Conversely, the inability to actively extend the affected fingers, actively grip and move a large tube, actively grasp and move a 2.5cm cube, or actively pronate/supinate the forearm with the elbow straight at all within thirty days post stroke demonstrated a statistically significant, moderate association with requiring moderate or more assistance at six months post stroke with washing and dressing oneself as tested by the self-care domain on the EuroQol-5D-5L. Partial ability to perform these tasks within thirty days post stroke was not significantly associated with self-care at six months.

Distal finger grasp, thumb adduction grasp, spherical and cylinder grasp were not significantly associated with self-care at six months, although thumb adduction grasp and cylinder grasp approached statistical significance. Perhaps a significant association would have been present with all four of these hand movements, given a larger sample size and thus greater power.

The ability to actively move the hand early after stroke, including grip ability and finger extension, is a known predictor of functional recovery after stroke at both the impairment and activity level [22,23]. Hand and non-synergistic, coordinated arm movements require some degree of CST function. CST integrity is an established predictor of upper limb motor recovery after stroke [11].

Affected hand and upper limb function are also associated with QOL which has been measured with various outcome measures including the EuroQol [5]. Few studies have looked at the specific domains of the EuroQol in the stroke population [24,25]. The findings from this study are unique as we showed an association between active affected hand function as well as non-synergistic and coordinated arm movements and the ability to wash and dress oneself, abilities critical for independence, at six months post stroke in moderate to severely affected individuals.

Prediction tools that combine clinical measures with neurophysiological and neuroimaging markers are deemed more accurate [3]. However, the tools and skills required to obtain neuroimaging and neurophysiological markers are not available in most clinics. Both the UEFMA and ARAT are reliable, valid, and widely used in the clinic and in research [9,10]. These outcome measures require little time to complete, are straightforward to use, require minimal equipment and would be easy to implement in the clinic and in the home setting. Completing the key items utilized in this study such as opening and closing the hand, touching the index finger with the thumb, pronating/supinating the forearm with the elbow straight, flexing extending the wrist with the arm straight and finally picking up objects and placing them on a low shelf would be even easier and faster to evaluate in the clinic than the entire tests. This would be valuable in the present clinical environment where there is an expectation to see large volumes of people in short amounts of time.

We propose that these easy to assess movements may be effective clinical tools to project independence with self-care in the long term in similar populations. This would allow rehabilitation clinicians working with stroke survivors in the early period post stroke to provide more realistic expectations and provide more efficient allocation of time and resources [4]. This was an exploratory study using a sample size of sixty. Thus, the findings would be more definitive if confirmed in a larger scale study.

## CONCLUSIONS

This exploratory study showed that the ability to actively move the hand, as well as perform non-synergistic and coordinated movements of the affected arm within thirty days post stroke was associated with greater independence with self-care at six months post stroke in persons with moderate to severe stroke.

## STUDY LIMITATIONS

As noted in the methods section, there were few participants with scores of four and five on the self-care domain of EuroQol-5D-5L at s months, so these scores were collapsed into a score of three. We did not measure CST function in this group-future studies would be needed to examine whether the presence of improvements in these hand and arm activities is accompanied by better functioning of the CST.

## Supplementary Material

Supplementary File

## Figures and Tables

**Table 1:** Score and related description for the self-care domain.

Score	Description
1	I have no problems washing or dressing myself.
2	I have slight problems washing or dressing myself.
3	I have moderate problems washing or dressing myself.
4	I have severe problems washing or dressing myself.
5	I am unable to wash or dress myself.

**Table 2:** Characteristics and outcome measure data of participants at baseline and outcome data at six months.

Baseline characteristics, mean (SD)	
Age	63.12 (10.99)
Sex	25 F and 35 M
Days post stroke at baseline	16.03 (6.37)
Total UEFMA score	31 (9.86)
Severity based on the initial total UEFMA score	9 severe and 51 moderate
Total ARAT score	16.46 (11.76)
Breakdown of itemized UEFMA scores, n (%)	
Mass finger extension	0 = 15 (25)
	1 = 34 (56.7)
	2 = 11 (18.3)
Mass finger flexion	0 = 6 (10)
	1 = 37 (61.7)
	2 = 17 (28.3)
Distal finger grasp	0 = 13 (21.7)
	1 = 37 (61.7)
	2 = 10 (16.7)
Thumb adduction grasp	0 = 26 (43.3)
	1 = 30 (50)
	2 = 4 (0.07)
Thumb to index finger grasp	0 = 31 (51.7)
	1 = 22 (36.7)
	2 = 7 (11.7)
Cylinder grasp	0 = 17 (28.3)
	1 = 28 (46.7)
	2 = 15 (25)
Spherical grasp	0 = 21 (35)
	1 = 29 (48,3)
	2 = 10 (16.7)
Forearm pronation/supination with the elbow straight	0 = 11 (18.3)
	1 = 48 (80)
	2 = 1 (0.02)
Wrist flexion/extension with the elbow straight	0 = 16 (26.7)
	1 = 39 (65)
	2 = 5 (0.03)
Breakdown of itemized ARAT scores, n (%)	
2.5 cm grasp	0 = 19 (31.7)
	1 = 13 (21.7)
	2 = 27 (45)
	3 = 1 (0.02)
Large tube grip	0 = 17 (28.3)
	1 = 13 (21.7)
	2 = 29 (48.3)
	3 = 1 (0.02)
EuroQol-5D-5L self-care outcomes at six months post stroke, n (%)	
Self-care	1 = 24 (40)
	2 = 23 (38.3)
	3 = 13 (21.7)

**Table 3:** Association between baseline UEFMA and ARAT scores and self-care from the EuroQol-5D-5L at 6 months.

UEFMA or ARAT item baseline score and number of participants who had that score, n	Proportion of people who achieved the baseline score on the UEFMA or ARAT item and their associated level on the EuroQol-5D-5L at 6 months post stroke	Fisher’s exact P value	Cramer’s V value P value
UEFMA Mass finger extension 2, n = 11	9/11 were independent	p = 0.006	0.363 p = 0.003
UEFMA Mass finger extension 0, n = 15	7/15 required moderate or greater assist	p = 0.006	0.363 p = 0.003
UEFMA Mass finger flexion 2, n = 17	12/17 were independent	p = 0.007	0.326 p = 0.003
UEFMA Thumb to index finger 2, n = 7	7/7 were independent	p = 0.018	0.327 p= 0.011
UEFMA Wrist flexion/extension with the elbow straight 2, n = 5	5/5 were independent	p = 0.011	0.343 p = 0.005
UEFMA Forearm pronation/supination with the elbow straight 0, n = 11	8/11 required moderate or greater assist	p = 0.001	0.43 p = 0.001
ARAT Large tube grip 0, n = 17	8/17 required moderate or greater assist	p = 0.020	0.339 p = 0.019
ARAT 2.5 cm grasp 0, n = 19	9/19 required moderate or greater assist	p = 0.026	0.338 p = 0.02

## Data Availability

The datasets used and/or analyzed during the current study available from the corresponding author on reasonable request. Request should be made to Jigna Patel, PT, DPT, MHS, PhD.
