# Supplementary material for: Association Between Upper Limb Impairment and Function Within One Month Post Stroke and Self-care at Six Months
Source: Int J Phys Med Rehabil. Author manuscript; Available in PMC 2026 Jan 28. (PMC12843392)
Supplement: Supplementary File [file NIHMS2126518-supplement-Supplementary_File.pdf]

International Journal of Physical Medicine & Rehabilitation

OPEN ACCESS Freely available online

Research article

Association Between Upper Limb Impairment and Function Within One Month Post Stroke and Self-care at Six Months

Jigna Patel[1*], Qinyin Qiu[1], Gerard G Fluet[1], Holly Gorin[1], Jennifer Gutterman[1], Kiran Karunakaran[3], Karen J Nolan[3], Emma Kaplan[3], Alma S Merians[1], Sergei V Adamovich[2]

[1]Department of Rehabilitation and Movement Sciences, School of Health Professions Rutgers, The State University of New Jersey, Newark, New Jersey, USA; [2]Department of Biomedical Engineering, New Jersey Institute of Technology, University Heights, Newark New Jersey, USA; [3]Kessler Foundation, West Orange, New Jersey, USA

Supplementary table 1: Means and SDs for baseline and six-month scores for each of the three training groups along with the ANOVA for each outcome and time

Outcome and time	EVR group mean (SD)	DVR group mean (SD)	DMUC group mean (SD)	ANOVA outcomes for between groups
UEFMA baseline	17 (13.94)	11.8 (9.68)	19.2 (13.51)	F(2,57)=0.731, p=0.486
UEFMA six months	41.15 (17.25)	43.7 (15.22)	43.1 (18.86)	F(2,57)= 0.037, p=0.964
ARAT baseline	33.55 (11.02)	30.05 (8.61)	30.55 (9.93)	F(2,57)=1.842, p=0.168
ARAT six months	53.95 (10.99)	54.95 (12.58)	53.95 (16.31)	F(2,57)=0.121, p=0.887

Correspondence to: Jigna Patel, Department of Rehabilitation and Movement Sciences, School of Health Professions Rutgers, The State University of New Jersey, Newark New Jersey, USA, E-mail: patel421@shp.rutgers.edu

Received: 14-Sep-2025, Manuscript No. JPMR-25-38720; **Editor assigned:** 16-Sep-2025, PreQC No. JPMR-25-38720 (PQ); **Reviewed:** 29-Sep-2025, QC No. JPMR-25-38720; **Revised:** 06-Oct-2025, Manuscript No. JPMR-25-38720 (R); **Published:** 13-Oct-2025, DOI: 10.35248/2329-9096.25.13.767.

Citation: Patel J, Qiu Q, Fluet G, Gorin H, Gutterman J, Merians A, Adamovich S, Karunakaran K, Nolan K, Kaplan E (2025). Association Between Upper Limb Impairment and Function Within One Month Post Stroke and Self-care at Six Months. Int J Phys Med Rehabil. 13:767.

Copyright: © 2025 Patel J, et al. This is an open-access article distributed under the terms of the Creative Commons Attribution License, which permits unrestricted use, distribution and reproduction in any medium, provided the original author and source are credited.
